# Scanning Electron Microscopy with Samples in an Electric Field

**DOI:** 10.3390/ma5122731

**Published:** 2012-12-11

**Authors:** Ludĕk Frank, Miloš Hovorka, Šárka Mikmeková, Eliška Mikmeková, Ilona Müllerová, Zuzana Pokorná

**Affiliations:** Institute of Scientific Instruments of the ASCR, v.v.i., Královopolská 147, CZ-61264 Brno, Czech Republic; E-Mails: hovorka@isibrno.cz (M.H.); sarka@isibrno.cz (Š.M.); eliska@isibrno.cz (E.M.); ilona@isibrno.cz (I.M.); zuza@isibrno.cz (Z.P.)

**Keywords:** scanning electron microscopy, slow electrons, low energy SEM, low energy STEM, cathode lens

## Abstract

The high negative bias of a sample in a scanning electron microscope constitutes the “cathode lens” with a strong electric field just above the sample surface. This mode offers a convenient tool for controlling the landing energy of electrons down to units or even fractions of electronvolts with only slight readjustments of the column. Moreover, the field accelerates and collimates the signal electrons to earthed detectors above and below the sample, thereby assuring high collection efficiency and high amplification of the image signal. One important feature is the ability to acquire the complete emission of the backscattered electrons, including those emitted at high angles with respect to the surface normal. The cathode lens aberrations are proportional to the landing energy of electrons so the spot size becomes nearly constant throughout the full energy scale. At low energies and with their complete angular distribution acquired, the backscattered electron images offer enhanced information about crystalline and electronic structures thanks to contrast mechanisms that are otherwise unavailable. Examples from various areas of materials science are presented.

## 1. Historical Introduction

**Figure 1:**
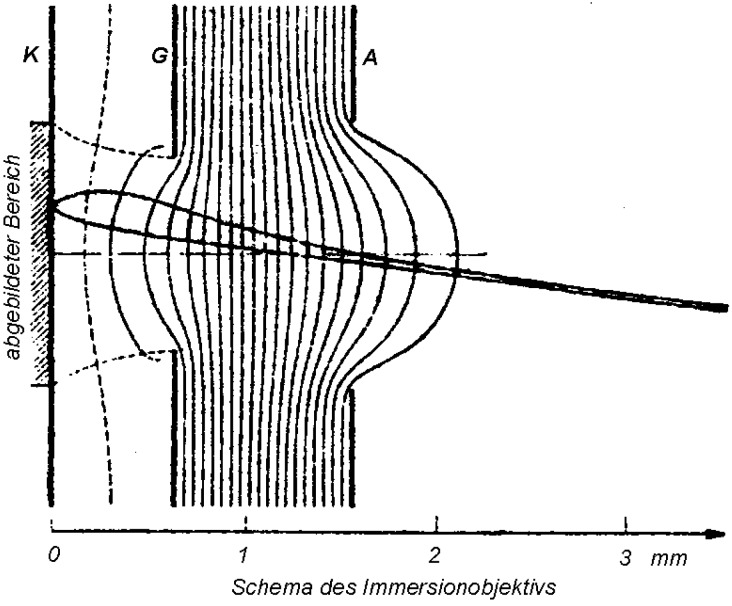
Scheme of the immersion objective lens reproduced from [2], in which the figure has been adapted according to the original in [1].

The idea of immersing the sample under observation in a strong electric field by means of electrons is one of oldest principles appearing in the development of electron microscopy. By the early 1930s, the so-called immersion objective lens was described [1] in which the sample served as the source of electrons and simultaneously as one of the electrodes of an electrostatic lens (Figure 1). Electrons emitted from an adequately excited surface of the sample were collimated and accelerated in an electric field between the sample surface and an anode placed above the sample. The complete assembly, with another electrode (grid) between the cathode and anode, is capable of focusing the emitted electrons into a real image. In 1941, a theoretical analysis [2] showed an image resolution proportional to the ratio of the initial emission energy and the energy acquired between the cathode and anode. This configuration later received the name emission electron microscope, and the immersion objective lens was called the cathode lens.

The production and employment of emission electron microscopes began in the early 1960s in Germany [3]. Surfaces under observation were either heated or bombarded with ions, photons or electrons. From our perspective, two turning points were important. The first was the design of the scanning electron microscope (SEM) with the sample negatively biased to reduce the landing energy of the primary electrons [4]. The second was a historical moment when the functional sample of the low energy electron (reflection) microscope appeared [5] with the sample surface bombarded by a coherent plane wave of very slow electrons so that the electron illumination column had to be similar to that of a SEM. This approach led to low energy electron microscopy (LEEM) instrumentation as one of the most successful pieces of equipment in surface science [6]. The combination of both these principles has been attractive for decades, and numerous designs of this kind have been published, as reviewed in [7]. Nevertheless, the published designs were usually not followed by results demonstrating acceptable imaging properties. The first scanned micrographs at very low energies were published in 1968 [8], though these were of rather low quality so no substantial progress was chronicled in the following two decades. Then, in the early 1990s, a program called scanning low energy electron microscopy (SLEEM) was initiated at the Institute of Scientific Instruments, Brno, Czech Republic. Analytical equations were derived for aberration coefficients of a two-electrode cathode lens in combination with a standard objective lens of the SEM [9], and a first series of micrographs of consistent resolution throughout the full energy scale down to 1 eV was published [10]. Since that time, the SLEEM method has been developed and demonstration experiments have authenticated its performance in various branches of materials science [11,12,13].

## 2. Motivation

Advantages of reducing the landing energy of the focused primary electron beam in the SEM were recognized at the very beginnings of the development of this kind of instrumentation. (Detailed discussion of this issue can be found in [6,11].) The obvious arguments include improved visualization of relief details, suppressed charging of samples and enhanced signal-to-noise ratio resulting from the increasing emission of secondary electrons (SE). Slow electrons penetrate only to shallow layers so the surface sensitivity increases while the lateral dimensions of the interaction volume diminish, meaning the relief is better represented and subsurface details on smooth surfaces are sharply imaged. The edge effect, consisting of overbrightening of steeply inclined facets and side walls, is greatly reduced and disappears completely below 500 eV. For a majority of materials the total electron emission yield is below 1 except for an interval between the “critical energies” where it surpasses the unit level. This interval of positive charging of the surface under bombardment extends between a few hundreds eV and a few keV. The positive charging is limited by retraction of a certain proportion of the emitted SE by the electric field generated above the charged surface.

At low energies, the crystalline information is enhanced, as for example, the grain contrast in polycrystals. The reasons for this phenomenon include the dependence of the generation and absorption of SE as well as of electron backscattering on crystal orientation, together with the increased influence of surface layers such as oxides also having their thickness orientation dependent. Angular variations in the backscattered electron (BSE) yield from crystals have been shown to dominate over those of the SE [14]. As we will see later, at very low energies the reflectance of electrons may indicate the local density of empty electron states above the vacuum level, *i.e.*, the electronic energy band structure characteristic for a particular crystal system and its orientation with respect to the incident electron beam.

In addition to the possibility of arbitrarily reducing the landing energy of electrons, the above-sample electric field is capable of collimating toward the optical axis the complete emission of BSE. Traditionally, the BSE emission has been acquired with a coaxial detector placed below the objective lens and held on ground potential. In this case the straight trajectories of the BSE impinge on the detector within a cone limited up to, say, a polar angle of 45° with respect to the optical axis, leaving the high-angle BSE unutilized. Experience has shown the high-angle BSE bearing significantly enhanced crystallographic information; this effect has been repeatedly verified, although clear explanation is still lacking. When immersing the sample in a strong electric field, we can easily control acquisition of the high-angle BSE already at only moderately reduced landing energy of electrons.

**Figure 2:**
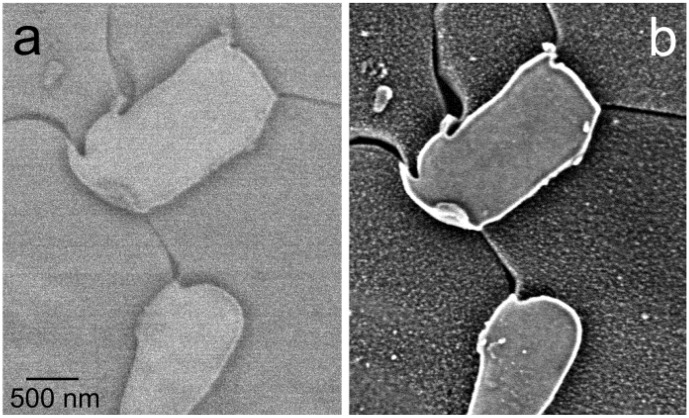
Mg_17_Al_12_ precipitates in the AZ91 alloy: (**a**) Standard imaging with backscattered electron (BSE) acquired between polar angles 10° and 45°; (**b**) Sample immersed in 450 V/mm field, polar angles 13° to 63° acquired. Landing energy is 2 keV for both frames.

**Figure 3:**
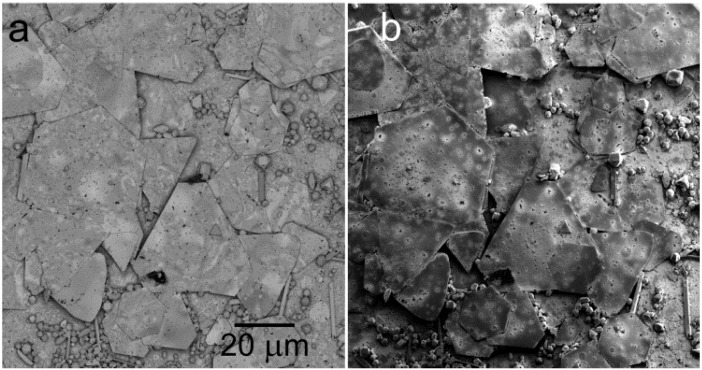
Microcrystalline Au deposited on glass. (**a**) Standard BSE imaging at 6 keV; (**b**) Sample in 1 kV/mm, landing energy of electrons 5 eV.

The above listed features of low-energy illumination and high-angle BSE detection are immediately demonstrated in the improved appearance of micrographs of a large variety of samples (Figure 2 and Figure 3). However, additional effects not available at traditional electron energies are connected with the electron wavelength approaching interatomic distances so that diffraction and interference phenomena can appear even in the reflected electron flux, as is the case in the low-energy electron diffraction (LEED) experiment. Amplitude addition within the primary spot (which is usually internally coherent) may generate fluctuations in the dark field image signal acquired with a coaxial detector from quasi-2D crystals in dependence on electron energy, *i.e.*, the diffraction contrast [15]. Similarly, interference contrasts can be expected in the divided-wavefront version on surface terrace steps as well as on ultrathin surface layers because of the divided-amplitude interference.

## 3. Electron Optical Aspects

We have cited the previously published calculation of the resolution obtained with electrons emitted from a sample placed in the cathode lens, namely, that it is proportional to the ratio of the initial and acquired energies. Simply stated: the lower the electron energy in the sample plane, the better the resolution. Considering this correlation in connection with the scanning electron microscope suffering from the opposite rule regarding the resolution *versus* energy dependence, we immediately recognize an interesting opportunity for radical improvement. We need only believe in the validity of the classical reciprocity theorem (see, e.g., [16]) declaring the identity of electron optical properties for both directions of passage through a column. Let us note that for imaging the samples placed in the cathode lens with an incident focused beam, we consider the ratio of the landing energy of electrons and their original energy in the illumination column.

Firstly, it is important to remember simple approximate relations defining the spot size for a conventional SEM with the sample in a field free space. The primary spot profile can be considered a convolution of the demagnified image of the gun crossover with discs of confusion of the basic aberrations, namely, the spherical, chromatic and diffraction aberrations. These contributions can, in their simplest form, be written as [11]:

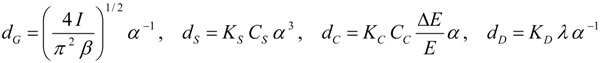
(1)
where *d_G_* is the demagnified crossover or the virtual source in the case of field emission guns; *d_S_*, *d_C_*, and *d_D_* are the discs of spherical, chromatic and diffraction aberration, respectively; *I* is the beam current; *β* is the gun brightness; *α* is the specimen-side angular aperture of the primary beam; *C_S_* and *C_C_* are the coefficients of spherical and chromatic aberration, respectively; Δ*E* is the energy spread of the primary beam; *λ* is the wavelength and *E* is the energy of electrons, and *K_S_*, *K_C_* and *K_D_* are numerical factors resulting from a model of spot formation. One summation rule combining these contributions simply adds their squares, which corresponds to a combination of mutually independent random variables with normal distributions. It is more realistic to take the disc sizes as diameters encircling some fraction of the total current, say, 50%. Then the summation rule is [17]:

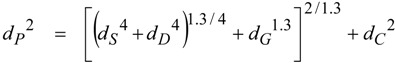
(2)
with *K_S_* = 0.18, *K_C_* = 0.34 and *K_D_* = 0.54. When substituting from Equation (1) to Equation (2), we get the final spot size *d_P_* as a function of the angular aperture *α* and electron energy *E*. The ultimate spot size is then obtained at the optimum aperture resulting from the condition ∂*d_P_*/∂*α* = 0. In this way we get the function *α_opt_* (*E*), which tends to *α_opt_* ~ *E*^1/4^ at low energies. Substituting this into the Equation (1), we get the strongest energy dependence as *E*^−3/4^, whose proportionality also holds true for the ultimate spot size. Therefore, a strong deterioration of the spot size, amounting to three orders of magnitude when changing from 10^4^ eV to 1 eV, prevents us from using true low energies in conventional SEM columns.

The reason for an abrupt extension of the spot at low energies is in the main aberration coefficients for conventional magnetic lenses, *C_S_* and *C_C_*, which are independent of energy. However, the combination of a two-electrode cathode lens with a standard focusing lens exhibits the aberration coefficients at low energies as follows [9]:

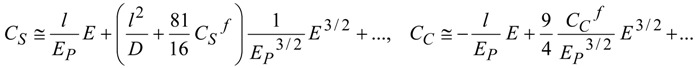
(3)
where *C^f^_S_* and *C^f^_C_* are the aberration coefficients of the focusing lens; *l* is the length of the electric field, and *D* is diameter of the anode bore. The proportionality of these final aberration coefficients to *E*, *i.e.*, their decrease with decreasing energy, reduces our ultimate result, the energy dependence of the spot size, to *d_P_*^min^~ *E*^−1/4^. This slope appears where higher terms in the Equation (3) become sufficiently negligible.

**Figure 4:**
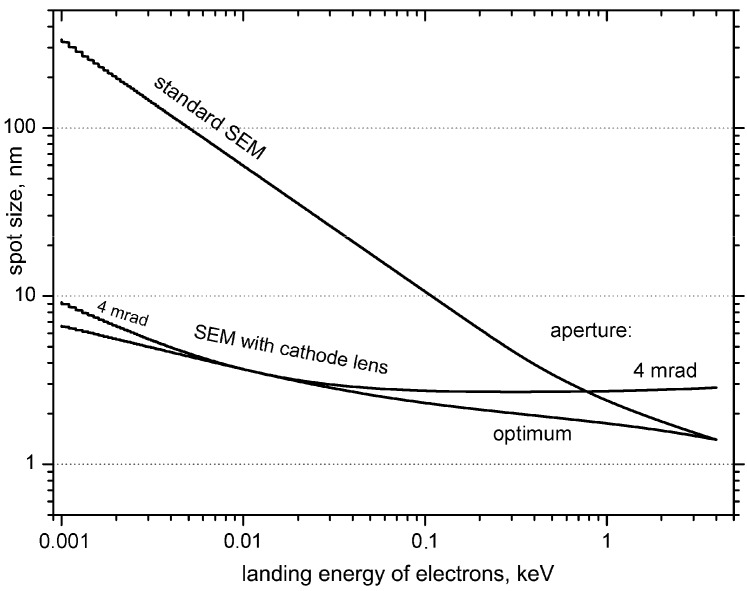
An example of the spot size data calculated from approximate analytical equations [9] for the objective lens defined with spherical and chromatic aberration coefficients 7.4 and 2.5 mm, respectively, primary beam energy 4 keV, working distance 4 mm and energy spread of the beam reduced with a monochromator to 100 meV. The gun brightness was 10^8^ Acm^−2^sr^−1^, the beam current was 10 pA, and the anode bore 1 mm in diameter.

In Figure 4 we compare the spot size *vs.* energy plot for a standard SEM configuration with that for a sample immersed in an electric field equal to the difference between primary and landing energies divided by the working distance. These curves are calculated for appropriate optimum apertures, which are energy dependent and hence would have to be adjusted separately for each landing energy, otherwise very simply controlled with the sample bias. In practice, we align the column at a certain primary energy and current, *i.e.*, at a certain angular aperture, and then vary the landing energy requiring only slight refocusing [18,19]. To assess this simplification, we have added to Figure 4 one more plot for a particular value of the aperture just before the cathode lens, namely a value that is the optimum at a landing energy of 10 eV. Differences between data for optimum and fixed apertures are obviously acceptable, at least below a few hundreds eV.

Implementation of the cathode lens principle is feasible in at least two versions. In both of them the sample is biased to a high negative voltage while the earthed anode is placed at a distance of few mm above the sample. To obtain quality images, we have to secure axial symmetry of the configuration. This includes a smooth flat surface of the electrodes, a circular bore in the anode well centered to the optical axis, and surfaces of the anode and cathode/sample as mutually parallel and normal to the optical axis as possible. Should the sample represent an adequately planar equipotential surface, it has to be not only flat and smooth but also large enough, *i.e.*, either of a size comparable to the cathode/anode distance or covered with a thin foil cap securing this relation. Experience has shown the requirement for the smoothness of the sample to be much less strict than we feared—even at units of eV, relief hollows and protrusions in units of micrometers are acceptable, with increased tolerance at higher landing energies. Naturally, the ultimate resolution requires ideally flat, if not polished surfaces under observation. The parallelism of the electrodes is best guaranteed with the specimen stage equipped with two independent, mutually perpendicular tilts.

**Figure 5:**
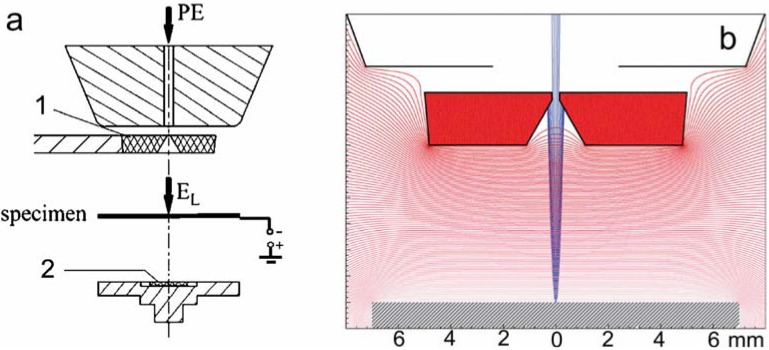
(**a**) Principal scheme of a cathode lens configuration with a negatively biased specimen, detector of reflected electrons 1 based on a coaxial bored single-crystal YAG scintillator and detector of transmitted electrons 2 based on a PIN diode; (**b**) Equipotential lines between the scintillator serving as the anode of the cathode lens and the specimen, together with trajectories of elastically reflected electrons; primary energy 6 keV, specimen bias−5.997 keV, landing energy 3 eV.

Signal electrons, accelerated and collimated in the electric field, move toward the anode. Two alternatives for their detection are described as follows. The anode bore may be large enough to let the signal “beam” through in a field free space where it can impact on a BSE/SE converter from which the secondary electrons are collected by a standard Everhart-Thornley detector [10]. In this regards, we should mention the outer diameter of the collimated signal bundle given as [11]:

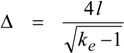
(4)
where *k_e_* = *E_P_*/*E_e_* with *E_e_* for the emission energy of electrons, usually considered equal or similar to the landing energy *E_L_*. This version is optimum for SEM configurations already equipped with a BSE/SE converter located above or inside the objective lens (OL). For traditional arrangements, in old types with no BSE detector or with a below-OL detector of the Robinson or Autrata types, the optimum solution is to insert just the below-OL detector of BSE. In order to tailor the balance between a reasonable restriction of the field of view and acceptable losses of lowest energy electrons (the specularly reflected ray in general), we can choose the central bore diameter in tenths of mm; a diameter of 0.3 mm has been used in multiple implementations with the scintillator disc made of single crystal YAG [11,20] (Figure 5).

As we will discuss later, at units of eV ultrathin films become transparent enough to be imaged in the STEM mode. The detector of transmitted electrons can be situated below the sample (Figure 5) under conditions similar to those for the detector of reflected electrons though simplified by the absence of the passage of the primary beam. The detector can be split into usual concentric rings for separated acquisition of the bright and dark field signals as well as the high-angle-annular-dark-field signal, but with dimensions of the rings corresponding to Equation (4).

## 4. Experimental Conditions

Thanks to its extreme surface sensitivity, the SLEEM belongs to methods relevant for analysis of a few uppermost atomic layers of samples and hence demands adequately clean and defined surfaces. This requirement is strict and difficult to meet, in particular from the standpoint of complexity and price of instrumentation. However, should the equipment be on a level corresponding to the nanostructures and nanotechnologies of today or even of tomorrow, securing ultrahigh vacuum (UHV) conditions and *in-situ* prepared, clean and defined surfaces is the only feasible way.

Experience has shown devices with standard vacuum chambers, *i.e.*, conventional SEMs, to be capable of producing quite acceptable micrographs at low and very low energies (in this review they are in Figure 24). Tolerance of vacuum conditions depends on a contrast mechanism under examination.

**Figure 6:**
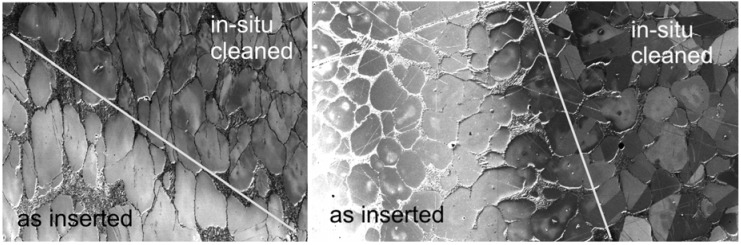
Margins of areas on steel samples, *in-situ* cleaned with a beam of argon ions in order to remove the oxide layer and other contaminations (3 keV beam incident for 60 min under an angle above 80° with respect to the surface normal); samples in 900 V/mm, landing energy 500 eV.

When using an UHV device, we normally clean the sample by rinsing in suitable solvents or even etchants but still, after loading it to the sample chamber, we find it covered with a thin adsorbed layer of air gases and hydrocarbons. Reactive samples are usually oxidized and after short illumination with electrons all surfaces become contaminated with a carbonaceous film produced by the decomposition of the adsorbed hydrocarbons. These usually diffuse intensively along the surface, giving rise to widely known black rectangles with noticeable frames. With decreasing energy of electrons, their depth of penetration diminishes faster than linearly so the spatial density of dissipated energy increases and simultaneously intensifies also the carbon contamination. The most intensive contamination is observed at 100 to 200 eV despite traditional expectations of the radiation damage simply fading out with decreasing energy of electrons. Individual phenomena embraced under the radiation damage disappear only below about 50 eV together with corresponding events of the inelastic scattering. The remedy consists either in outgassing the loaded sample before observation by means of moderate heating to about 100 °C, or in sputtering off the surface layer with an ion beam (Figure 6). For the bombardment, inert gas ions are suitable at a low energy and glancing angle. Under standard vacuum conditions we face not only the presence of the layer loaded together with the sample but also adsorption of gases from the microscope chamber so we have to cope with formation of a surface coating that submerges or even eliminates certain types of contrast. Partial help is available from plasma cleaners, cooled traps or ions available in dual beam devices.

All except one of the micrographs presented hereinafter have been acquired in a dedicated SLEEM device working under UHV conditions and equipped with a facility for *in-situ* cleaning of surfaces with an Ar ion beam. The column of the microscope is an all electrostatic two lens column providing ultimate resolution of about 10 nm at 10 keV and 25 nm at 10 eV.

## 5. Experimental Results

### 5.1. Traditional SEM Contrasts

**Figure 7:**
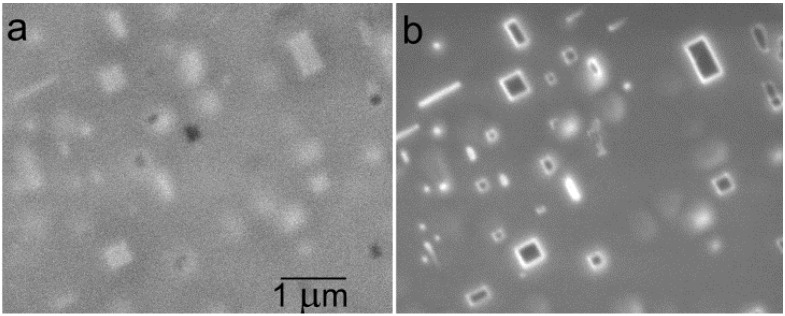
Precipitates in the Al-1.0 mass% Mg_2_Si with 0.4% excess Mg alloy, heated for 60 min at 848 K, quenched in chilled water and age-hardened for 60 min at 673 K. (**a**) Standard BSE image obtained at 10 keV with a below-OL coaxial detector; (**b**) Sample immersed in 1.2 kV/mm field, landing energy 1500 eV.

As we have stated, among factors motivating us to introduce the SLEEM method, several phenomena exist complicating the standard SEM operation. One is the size of interaction volume achieving units of micrometers at tens of keV. Although the SE are emitted only from a shallow subsurface layer the thickness of which is governed by the rate of absorption of hot electrons and is therefore significantly thicker for nonconductors but still not exceeding 10 to 20 nm, the BSE can escape from substantial part of the interaction depth that approaches 50% of this depth at low keV [21]. Moreover, the lateral diffusion of primary electrons extends the information spot dimension far behind the primary spot dimension so the “real” resolution is generally much worse than the nominal one [22]. We do well to remember that samples usually used for demonstrating the guaranteed resolution are composed of islands of a thin layer of a heavy element, deposited on a light element substrate so that edges of the islands are imaged preferably with the primary spot size electrons. The “illumination” of surface islands with the BSE coming from depth, which produce the SE2 signal [23], is so diffuse that it does not adulterate the image, at least at tens of keV. At and below the units of keV, the SE2 contribution, when convolved with the SE1 signal produced by the primary beam, may already worsen the figure. However, far more critical is the imaging of tiny 3D objects like various precipitates, buried to flush with the surrounding surface. As we see in Figure 7, even a moderate retardation of the illuminating electrons secures the BSE micrographs which are significantly improved both in contrast and resolution.

A further phenomenon to note is the edge effect, *i.e.*, overbrightening at steeply inclined terraces that hampers, e.g., measurement of critical dimensions on semiconductor structures. The effect appears when the penetration depth of primary electrons is larger than the escape depth of signal electrons, in which case the glancing illumination of inclined facets provides an additional emitting surface. The effect diminishes with decreasing energy and fully disappears approximately at an energy for which the SE emission from the sample achieves its maximum at hundreds of eV [24].

We have briefly discussed the problem of charging up nonconductive samples with a charge difference between incident and outgoing electrons. Between critical energies the positive charging to only moderate surface potential of few volts is usually acceptable [25]. An optimum solution is to use the second (higher) critical energy, which is spontaneously set in the charging-up process so when we intentionally preset this landing energy with the sample bias, no charging take place. The critical energies are conveniently identified and adjusted using the sample bias in the SLEEM mode [26,27].

Finally, we comment on the material contrast, *i.e.*, the direct proportionality between the BSE yield and the mean atomic number of a sample. Reliable proportionality of this kind fades out below, say, 5 keV even for surfaces *in-situ* cleaned with ions [28]. However, at low energies one may tailor the landing energy so that the BSE contrast is enhanced between particular sites of only small difference in the mean atomic number.

### 5.2. Grain Contrast

When observing a polycrystalline target under standard vacuum conditions, we usually have the sample covered with a thin oxide film of a thickness dependent on the grain orientation and reactivity of the sample material. At low energies the growing surface sensitivity generates a rather distinctive grain contrast of surface coatings, combined with the channeling contrast responding to the presence and widths of channels between atomic columns, which enable electrons to penetrate in depth, hence reducing the backscattering. Cross-sections of the channels depend on the crystal system, its spatial orientation and the angle of impact of the primary electrons.

With a really clean surface we have a pure channeling contrast which may be, however, influenced by surface reconstruction that shifts the surface atoms off their regular positions in the atomic columns. One possible way of distinguishing between both situations will be presented in Section 5.4.

We have referred to the BSE signal exhibiting larger yield fluctuations with the illumination angle than those of the SE [14]. The same study presents these fluctuations as growing with decreasing energy of electrons, which fully fits our practical experience. However, the SLEEM practice has shown the grain contrast influenced also by the angular range of the detector acceptance. When placing the sample in an electric field collimating the high angle BSE toward the optical axis and onto the below-OL detector, we get significantly enhanced grain contrast [29,30]. This contrast can be taken as conveyed by the BSE together with fast SE only because the slowest electrons, in particular the SE emitted at units of eV, partially or even fully escape detection through the detector bore.

**Figure 8:**
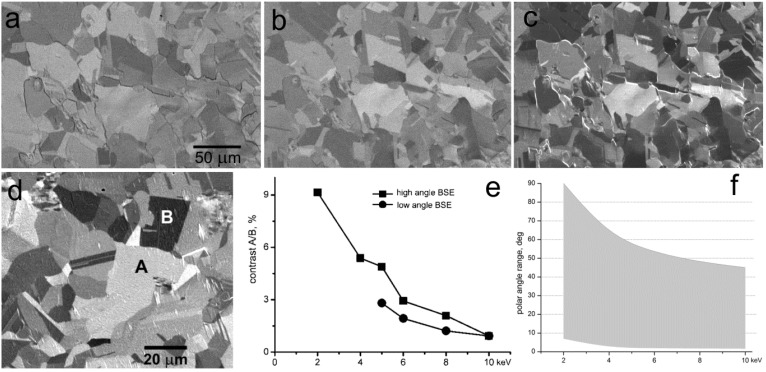
Polycrystalline Cu. (**a**) Standard BSE imaging at 10 keV; (**b**) Standard BSE imaging at 5 keV; (**c**) Sample in 900 V/mm, landing energy of electrons 5 keV; (**d**) Two grains chosen to measure the grain contrast (micrograph taken at 2 keV with BSE emitted between polar angles 8° and 90°); (**e**) The landing energy dependence of the grain contrast for the standard BSE imaging (low angle BSE) and for the primary electrons decelerated from 10 keV (high angle BSE); (**f**) The polar angle range accepted for the detection in the “high angle BSE” mode.

Measurement of the grain contrast has been performed on an *in-situ* cleaned, well relaxed polycrystalline Cu, as shown in Figure 8. Contrast between the marked grains was measured at various landing energies, first without any electric field applied to the sample and then at the same landing energies adjusted at a constant primary energy by means of the growing sample bias. With the increasing electric field, not only the landing energy diminishes but also the maximum polar angle accepted with the detector expands. In this way we can compare the clean effect of the decreasing energy with the same progression combined with increasing emission angle. Obviously, both influences mutually magnify.

**Figure 9:**
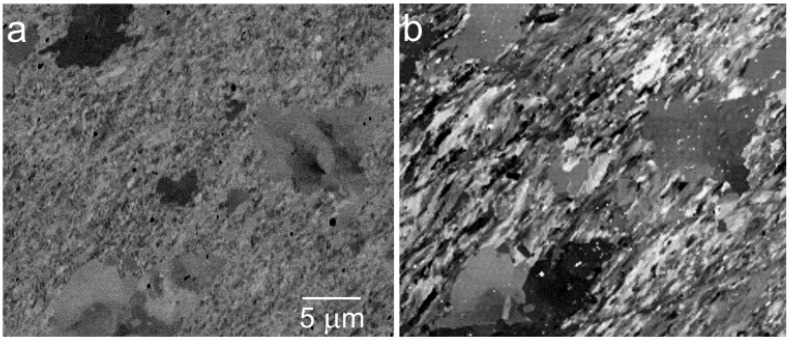
Ultrafine grained Cu prepared with the equal-channel-angular-pressing (ECAP) procedure and partially relaxed at 300 °C for 6 min in a vacuum. (**a**) Standard BSE image at 6 keV; (**b**) Sample in 1 kV/mm, landing energy 25 eV.

**Figure 10:**
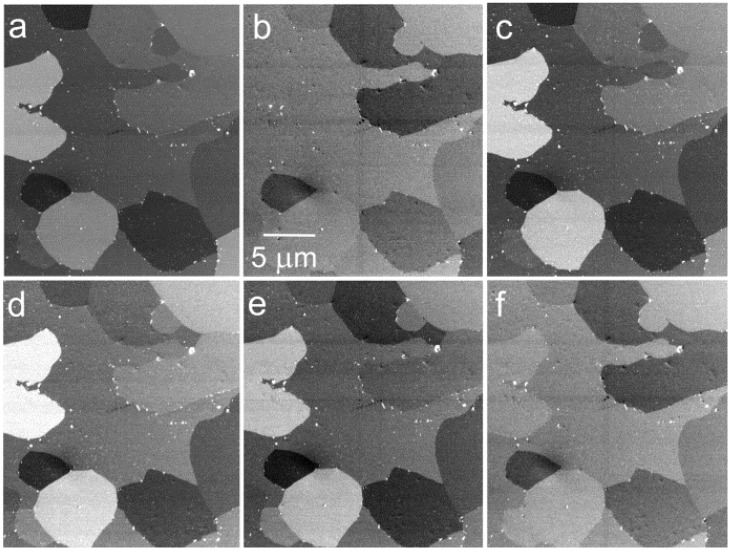
The Al-Mg-Si alloy with Mg_2_Si precipitates, electric field of 1 kV/mm, landing energies of electrons as follows: (**a**) 470 eV; (**b**) 420 eV; (**c**) 370 eV; (**d**) 270 eV; (**e**) 170 eV; (**f**) 70 eV.

The channeling contrast, obtained at low energies and enhanced via acquisition of the high angle BSE, works well in numerous application tasks. As distinct from the traditional electron backscatter diffraction (EBSD) method, this kind of imaging does not offer any straightforward way to identify absolute crystal orientations of the grains. Its advantages include high spatial resolution (see Figure 9), available as a result of the interaction volume much smaller than required by the EBSD (energy above 10 keV or at least above 5 keV, and sample tilt about 70°), and fast data acquisition. A series of micrographs of larger grains, taken at energies varying over a broader interval and showing rich variations in mutual contrasts including inversions (Figure 10), indicates possible identification of the orientation upon energy dependence of the mean signal over the grain in question.

**Figure 11:**
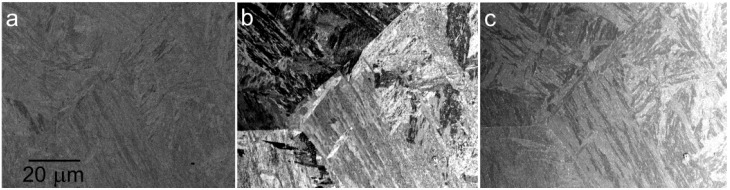
The CB2 steel of an acicular martensite structure. (**a**) Standard BSE imaging at 6 keV; (**b**) Sample in 900 V/mm field, landing energy 500 eV; (**c**) Sample in 1 kV/mm, landing energy 50 eV.

**Figure 12:**
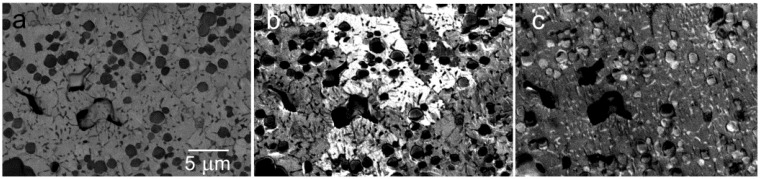
The CPM15V steel made by powder metallurgy, showing austenitic grains, acicular martensite and carbide precipitates. (**a**) Standard BSE imaging at 6 keV; (**b**) Sample in 900 V/mm, landing energy 500 eV; (**c**) Sample in 1 kV/mm, landing energy 50 eV.

One interesting phenomenon has been observed with certain samples, for example, special steels of a rather complicated structure. Contrast between structural details of these samples increases with the decreasing energy of electrons down to between 500 and 1000 eV but then it weakens again, at least regarding particular details. The effect is illustrated in Figure 11 and Figure 12.

Both micrograph series, sampled in Figure 11 and Figure 12, provide the highest contrasts of planar features around 500 eV. With the primary energy of 6 keV, at 500 eV, the BSE are detected up to their emission angle of 90°. The central bore of 0.3 mm in diameter fully absorbs electrons below 1.35 eV and 3 eV electrons are absorbed to 50% so the SE contribution is severely reduced. The reason for the observed behavior may be sought at a different information depth, which can be estimated two to three times shorter at 50 eV with respect to 500 eV, not exceeding 0.5 nm, *i.e.*, one or two atomic planes [31]. Thus, the fading contrast may indicate the responsible structure features not rising to the very surface.

**Figure 13:**
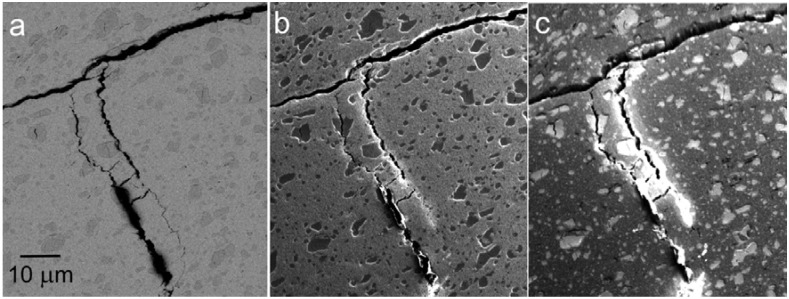
The X210Cr12 steel deformed with severe torsion and pressure (the TT deformation) at 350°C, showing vanadium-chromium precipitates. (**a**) Standard BSE image at 7 keV; (**b**) Sample immersed in 1 kV/mm field, landing energy 1 keV; (**c**) Sample immersed in 1.2 kV/mm field, landing energy 15 eV.

Precipitates are specific structural details usually present at high density with identical crystalline structure and even identical orientation with respect to the surface plane. In this case, they are imaged at the same average brightness but along the energy scale they can vary or even invert their contrast with respect to the matrix (Figure 13). Less frequent are precipitates of multiple structures or at least different orientations; in this case we can tailor energy of electrons to optimum contrasts between species (see Figure 7).

### 5.3. Mapping of Residual Strains

Grain contrast provides a tool for examining polycrystals as regards various statistical data such as, texture, grain sizes and shapes, orientation of grain boundaries, *etc.* However, insight into technological processes requires additional information, in particular information about residual strains inside grains or crystals in general. The EBSD method is capable of detecting the strain upon displacement of certain crystallographic features like the zone axes [32]. Analysis of the EBSD pattern from a strained crystal requires revealing shifts far less than one pixel. The strain tensor can be extracted by processing data from four independent measurements of positions in the EBSD patterns. An example of mapping the strain in polycrystalline nickel, presented in [32], shows the strain distribution decomposed to the rotational, shear strain and normal strain components and represented with contrast variations inside grains up to about 1%.

**Figure 14:**
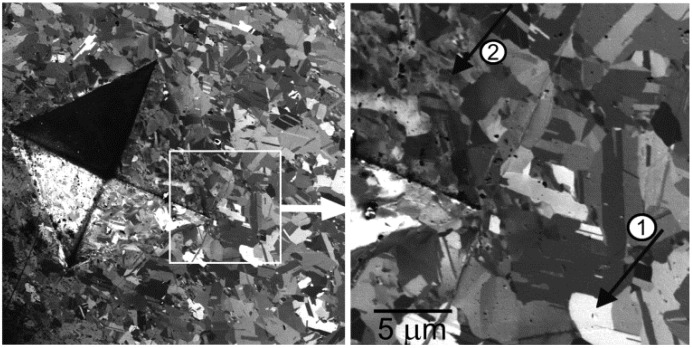
Micrograph of a Vickers indent in polycrystalline Cu with the magnified part comprising relaxed grains with a constant image signal (arrow 1) as well as deformed grains with internal signal variations (arrow 2). (scanning low energy electron microscopy (SLEEM) mode at 500 eV, which is capable of visualizing the strain-caused signal variations inside crystal grains on the indent wall.)

It would be highly desirable to image the strain directly, although possibly without measuring the strain tensor. With the SLEEM micrographs taken at hundreds of eV on deformed polycrystalline targets, we observe signal variations inside grains providing contrasts on similar or even higher level than that mentioned above. In the EBSD, the strain is detected on differences between patterns from a strained area and from an unstrained region of the crystal. In Figure 14 we can compare grains in the unstrained area far enough from the indent with grains on the indent wall where some residual strain is undoubtedly present. Signal variations inside deformed grains are obvious. Let us note appearance of the inclined indent wall undamaged with the above-sample electric field, with only slightly reduced depth of focus. This corresponds to a moderate beam deceleration from 6 keV to 500 eV.

**Figure 15:**
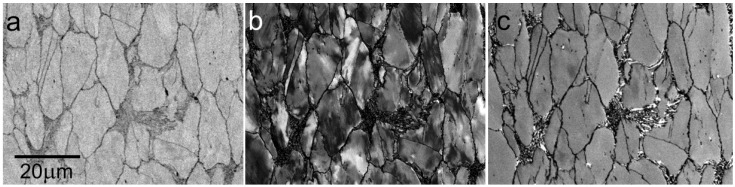
The X210Cr12 steel heated to semi-solid state at 1265 °C, deformed 2:1 at 800 °C and cooled. (**a**) Standard BSE image at 6 keV; (**b**) Sample immersed in 900 V/mm, landing energy 500 eV; (**c**) Sample immersed in 1 kV/mm, landing energy 50 eV.

**Figure 16:**
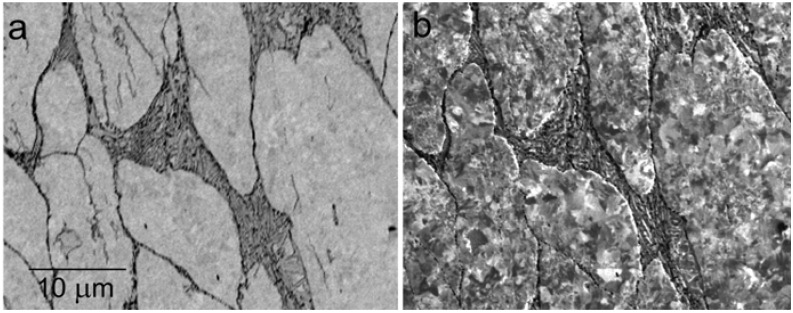
The X210Cr12 steel processed with the semi-solid technology (see Figure 15) and annealed at 500 °C for 60 min. (**a**) Standard BSE image at 8 keV; (**b**) Sample immersed in 900 V/mm, landing energy 500 eV.

A series of experiments has been performed with severely deformed steel passed through the semi-solid state. The structure shown in Figure 15 is composed of austenitic grains surrounded with eutectic and provides high strains visible inside the grains. After annealing, the strain is relaxed and the austenite fall into very fine grains without an apparent dominating orientation (Figure 16). The interesting detail cited above is the maximum strain contrast observed at 500 eV but diminishing at lower energies, similarly as the grain contrast that we observed in Figure 11 and Figure 12 and interpreted as an effect connected with variations in the information depth.

Experiments with imaging the strain contrast by means of slow electrons and with complete acquisition of angular distribution of the BSE are at an early stage so no procedures for quantification of the strain are available as yet.

### 5.4. Very Low Energy Reflectance

It is known from the LEED experiments that before any nonzero diffraction spot emerges we observe a so-called energy band contrast at the outset of the current *versus* voltage dependence for the specular (00) spot. The explanation is that sufficiently close to the vacuum energy level the crystal potential substantially influences the parabolic bands of free electrons and hence the unoccupied electron states above the vacuum level acquire dispersions that are characteristic of the crystal system and its spatial orientation [33]. Thus, reflectance of such slow electrons can be considered inversely proportional to the local density of states coupled to the incident electron wave [34]. Simply stated, electrons can penetrate into the crystal only when adequate Bloch states are available for the particular wave vectors; otherwise they are reflected. Naturally, observation of this phenomenon requires not only an atomically clean but also a reconstructed surface without contaminations or amorphous overlayers [35]. An additional condition is the landing energy below 25 or 30 eV where absorption of hot electrons is sufficiently low, *i.e.*, the electron lifetime is long enough for the reflection process to pass [36].

When measuring the reflectance of very slow electrons from a clean crystal surface *versus* the landing energy, we obtain a curve characteristic for the crystal system and its orientation with respect to the direction of electron impact. Thus, the reflected current can serve as an image signal providing information similar to that obtained from the channeling contrast at higher energies, among others the visualization of grains in polycrystals.

**Figure 17:**
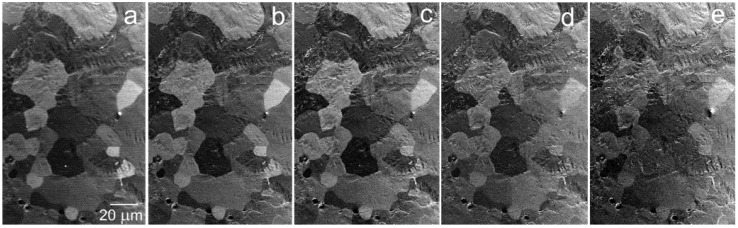
Polycrystalline Al, sample immersed in 1 kV/mm, landing energies of electrons as follows. (**a**) 2 eV; (**b**) 4 eV; (**c**) 7 eV; (**d**) 12 eV; (**e**) 20 eV.

Pilot experiments have been made on aluminum. The surface of aluminum has proven to be very difficult to prepare as really clean and free of any damaged layer. Figure 17 shows a series of micrographs of clean polycrystalline aluminum, recorded under UHV conditions but still not appropriate for the reflectance measurement. This is indicated with moderate changes and only exceptional inversions in the mutual contrasts along the energy scale. The grain contrast observed in this case should be primarily ascribed to surface barrier properties, possibly together with a thin oxide layer of thickness dependent on the grain orientation.

**Figure 18:**
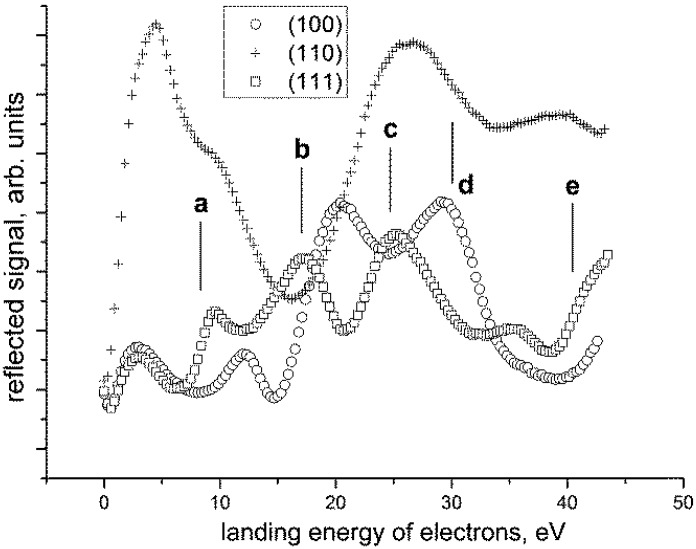
Reflectance *vs.* energy plots for three basic orientations of *in-situ* cleaned single crystals of aluminum. Five energy values, labeled **a** to **e**, refer to Figure 19.

A successful series of experiments [37] have been performed first on three single crystals of orientations (100), (110) and (111) and then on pure polycrystalline aluminum samples. After rinsing in methyl alcohol prior to loading, the samples were *in-situ* cleaned in three cycles. Each cycle consisted of argon ion sputtering to remove the oxide layer, followed by electron bombardment heating to 400 °C for 1 min. The sputtering was made for 15 min with a 1 keV argon ion beam under a 5° to 9° angle of incidence with respect to the sample surface, with the stationary spot of the ion beam and current of about 40 nA flowing through the sample. For checking the cleanliness of the sample, the most efficient method has proven to be the presence or absence of the raster “burn marks”, *i.e.*, black rectangles created on the surface due to prolonged scanning with the electron beam—on a well cleaned surface no burns appear even after hours of scanning. In Figure 18 the reflectance curves for all three orientations of the Al single crystals are compared. Obviously, the curves are mutually different enough to characterize the crystal orientation.

**Figure 19:**
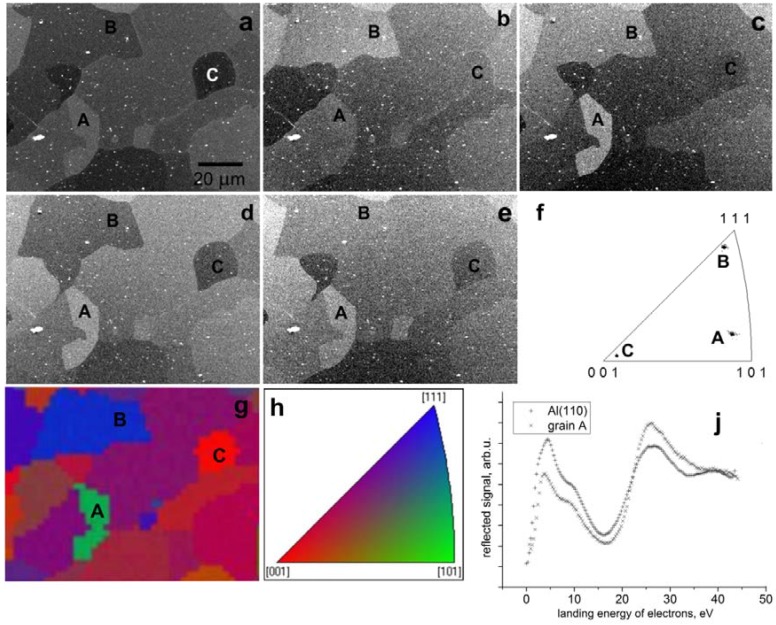
*In-situ* cleaned polycrystalline aluminum with three grains labeled A to C, imaged at very low energies. (**a**) 7.8 eV; (**b**) 17.4 eV; (**c**) 24.6 eV; (**d**) 30.9 eV; (**e**) 40.8 eV; (**f**) EBSD inverse pole map showing grains A to C; (**g**) EBSD mapping of the same area. (**h**) Color coding triangle; (**j**) Comparison of reflectances from the (110) single crystal and grain A.

Comparing curves acquired on the single crystals, we can identify electron energies at which the reflectances may be sufficiently different in order to distinguish the corresponding crystal orientations even in polycrystalline targets. Should just these three orientations be identified, the simplest approach is to apply energies at which the particular grain orientation provides the lowest or highest signal of all. This is demonstrated in Figure 19 on micrographs acquired on a clean polycrystalline Al with three grains labeled A, B and C that were verified with the EBSD analysis as being not far from the previously cited orientations. Angular differences were 10.7° between grain B and the orientation (111), 4.1° between the grain A and (101), and finally 8.1° between C and (001). Micrographs in Figure 19, taken at five energies labeled in Figure 18, exhibit contrast relations fully corresponding to relations among the reflectances. On the other hand, the offset of the reflectance curve for the grain A from that of the (101) single crystal is sufficient to recognize the angular difference [37].

The possibility of observing the energy band structure on the LEED data has been known for decades. However, when combining this mechanism of electron scattering with the SLEEM principle, enabling one to image the target surface at high resolution even when using such slow electrons, we get an added value in visualizing under perfect localization the physical parameters like the electron dispersion relations above the vacuum level. Extension to the microscopic mode addresses heterogeneous crystalline materials, *i.e.*, deformed crystals and polycrystals.

### 5.5. Dopant Contrast

Semiconductor structures represent one of hottest family of samples to be examined with the SEM and hence also with the SLEEM. The very first SEM micrograph showing the dopant contrast was published as early as 1967 [38]. The prospective of the SEM for examination of doped semiconductors was presented in 1995 [39] and in the years following numerous publications were dedicated to the topic (e.g., [40]). A brief summary should mention the p-type patterns generally brighter than n-type ones, linear (but not very reproducible and reliable) dependence of the image intensity on logarithm of the dopant concentration, and optimum energy around 1 keV for mappings with the secondary electron signal. Among models explaining the contrast mechanism (e.g., [41]) properties of the surface potential barrier are most often considered, namely the barrier height, which is governed by the dopant type and density, and so-called patch fields above the sample surface. Energy filtering of the SE signal has proven useful for enhancement of the contrast and its more accurate quantification [42].

The first study of the dopant contrast with the SLEEM method [43] compared the image signals from p^+^, n^+^ and n-type regions down to 15 eV landing energy and proposed explanation based on contacts between the semiconductor and the surface graphitic contamination. Detailed SLEEM study of a planar p/n structure [44] confirmed the SE emission as the contrast carrier throughout a broad energy range but pointed out the influence of surface coatings and contaminations. The SLEEM signal was found enhanced with respect to that available in the SEM and explained as a consequence of the external electric field of the cathode lens, contributing to the formation of subsurface space charge layers. The role of subsurface fields was confirmed with experiments on structures covered with very thin metal layers of different work functions [45]. A strong impact of the carbonaceous contamination on the dopant contrast was examined in dependence of the electron dose and vacuum conditions [46]. Critical survey of experimental data as well as contrast mechanism models can be found in [47]. The photoemission electron microscopy study produced a proposal to explain the observed phenomena with p/n differences in absorption of the hot electrons on their move toward the surface, existing due to a different energy threshold for generation of the e-h pairs [48]. The first detailed study of the n-type patterns on the p-type substrate [49] produced data previously not available, which we discuss below.

**Figure 20:**
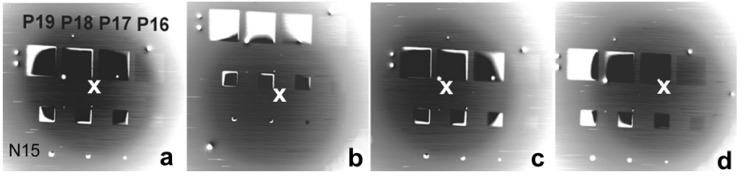
P-type doped rectangles on the n-type Si substrate (Px or Nx means the dopant density of 10^x^ cm^−3^), sample immersed in 1 kV/mm field, landing energy of electrons 1.6 eV. Black sites correspond to the total reflection of primary electrons in the detector bore while from white sites the electrons are totally reflected on the detector surface. Layout moves with the sample position slightly varied between frames: (**a**), (**b**), (**c**), and (**d**) show different positions of the pattern group with respect to the optical axis indicated with the cross.

Finally, we consider an alternative contrast mechanism that appears on p-type patterns illuminated with electrons in the range of units of eV [50]. Experiments were made with the cathode lens configuration according to Figure 5 and provided micrographs with extremely high contrasts appearing even inside the p-type patterns (Figure 20). The proposed explanation was based on charging the p-type patterns to about −1 V with injected electrons, which surface charge was sufficient for total reflection of primary electrons either on the detector surface or to its bore. Such a high contrast may be utilized when measuring the critical dimensions in semiconductor structures.

The above cited studies drew attention chiefly to the high sensitivity of the dopant contrast to cleanliness of the surface and to the history of the sample as regards treatments before and after loading in the SEM chamber. Low reproducibility of the quantitative data was obviously due to measuring each dopant concentration on a separate sample of a not exactly defined surface status. So as to avoid differences in the history of samples, a dedicated sample was manufactured with the dopant density varying within a broad range of four orders of magnitude along groups of strips and rectangles (see Figure 20) [50]. The samples were available in both n/p and p/n combinations.

**Figure 21:**
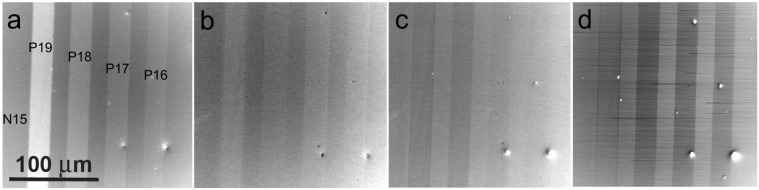
P-type doped stripes on the n-type Si substrate (Px or Nx means the dopant density of 10^x^ cm^−3^), sample immersed in 1 kV/mm field, landing energy of electrons as follows. (**a**) 1 keV; (**b**) 120 eV; (**c**) 18 eV; (**d**) 2.5 eV.

**Figure 22:**
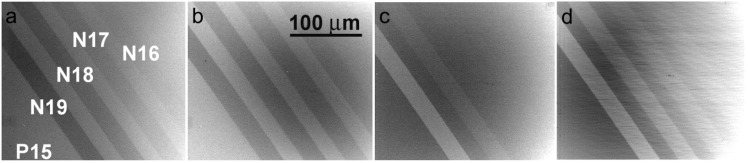
N-type doped stripes on the p-type Si substrate (Nx or Px means the dopant density of 10^x^ cm^−3^), sample immersed in 1 kV/mm field, landing energy of electrons as follows. (**a**) 2.5 keV; (**b**) 1 keV; (**c**) 100 eV; (**d**) 2.3 eV.

Figure 21 and Figure 22 illustrate the SLEEM micrographs taken over the energy range from units of keV to units of eV. Around 1 keV the p-type is always brighter than the n-type of silicon in accordance with previous experiments and, moreover, the signal intensity is reliably and reproducibly proportional to the dopant density of the p-type as well as of the n-type, although not exactly to logarithm of the density. However, with decreased landing energy of electrons, the contrast first loses its proportionality to the dopant density, then inverts but restores the density dependence and finally again loses the proportionality. This series of phenomena is common to both combinations of the p- and n-types; only energies at which the contrast transforms occur are slightly different. We admit that no model explaining these phenomena is available as yet. The samples were etched in buffered HF but no *in-situ* treatment was performed so that very thin oxide and hydrocarbon layers may form on surfaces before their loading in UHV and participate on phenomena observed.

### 5.6. Transmission Mode

Examination of free standing thin films with transmitted electrons is traditionally performed with the directly imaging transmission electron microscope (TEM) by means of fast electrons. Typical energies of the TEM varied during the history from tens of keV up to units of MeV and then back to some 200 to 400 keV as the most frequent values at present. Use of very high energies was motivated with efforts to improve resolution by reducing the diffraction aberration. With onset of the Computer-aided Design (CAD) programs for design of lenses and columns that provided lower geometrical aberrations, the MeV range was abandoned, while later the aberration correctors made possible the recent TEM solutions operated in tens of keV. On the other hand, efforts to increase the image contrast led to the opposite extreme, *i.e.*, the TEM at units of keV [51].

Scanning transmission electron microscopy (STEM) as the traditional counterpart to the TEM is performed with relatively rare dedicated instruments, aberration corrected and reaching resolution in tens of pm [52,53], and also with attachments to the TEM devices. More recently, also SEM devices have been equipped with STEM attachments operated at energies up to 30 keV with thinner samples and the advantage of multiple signals simultaneously acquired in the SEM is balanced with absence of the atomic resolution of the TEM.

The inelastic mean free path (IMFP) of electrons in solids exhibits more or less uniform energy dependence with a global minimum falling below 1 nm at about 50 eV [31]. Below that minimum, the IMFP rapidly increases again and at the units of eV it achieves values corresponding to units, if not tens of keV. This circumstance increases the chances for the STEM to be operated at units of eV but the elastic mean free path (EMFP) keeps shortening down to the range where the phenomena described in Section 5.4. start to take place. Strong material dependence of the electron reflectance makes application of the very low energy STEM (VLESTEM) sample specific.

**Figure 23:**
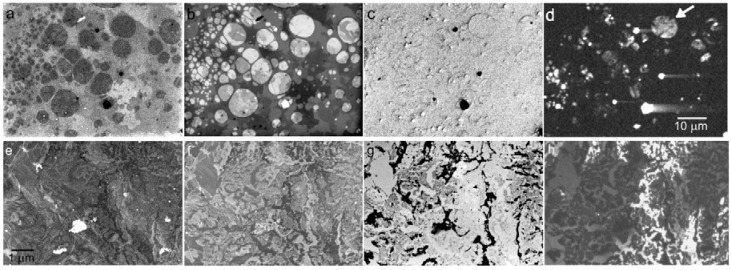
Micrographs of two graphene samples in combined reflection and transmission modes. (**a**) sample A, BSE at 3 keV; (**b**) sample A, TE at 3 keV; (**c**) sample A, BSE at 4 eV; (**d**) sample A, TE at 4 eV; (**e**) sample B, BSE at 5 keV; (**f**) sample B, BSE at 1 keV; (**g**) sample B, TE at 500 eV; (**h**) sample B, TE at 20 eV. (Sample A was purchased as the CVD graphene^TM^, sample B was provided by K. Novoselov, University of Manchester.)

Pilot experiments with the VLESTEM mode [54] revealed high thickness contrast on a 3 nm Au foil and pointed out that the electric field in the sample vicinity accelerates also the SE released near the bottom surface of the sample, generating in this way an “incoherent” contribution to the transmitted electron (TE) signal and apparently increasing the sample transmissivity to above 100% at the landing energies in hundreds of eV. Fortunately, in this energy range the SE are collimated to near the optical axis so their impact may be restricted to the bright field detector only, while the transmitted electrons should be acquired in the dark field channel as a pure signal [55]. Figure 23 shows two series of micrographs taken on two different graphene samples so that reflected and transmitted signals are recorded simultaneously.

Figure 23 shows that typical graphene samples consist of micrometer size, mutually overlapped flakes. While at higher energies some parts seem to be empty holes, at very low energies we show sites of very thin layers at a high contrast. In these windows (see the arrow) we expect again the overlapped flakes, possibly including true single layer graphene—this would have to be confirmed e.g., with the Raman spectroscopy.

The VLESTEM method is in its initial phase so only few preliminary results are available but prospects include the possibilities of detailed examination of any 2D crystals. Implementation of multiple channel detectors with separation both in polar and azimuthal angles is desirable.

### 5.7. Thin Surface Coatings

**Figure 24:**
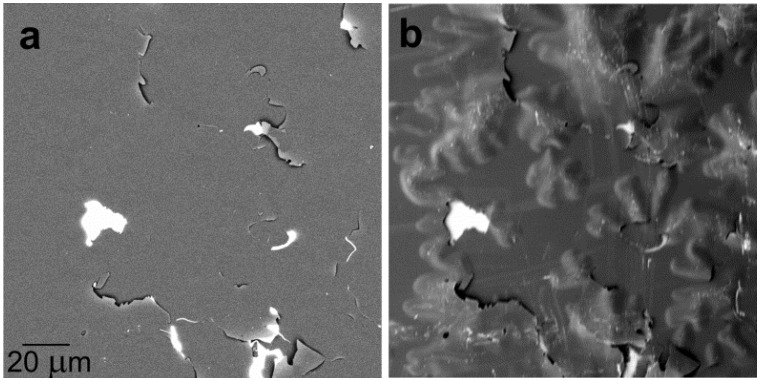
Delaminated 200 nm CN_x_ layer, RF magnetron sputtered on silicon (100), sample in 1 kV/mm field. (**a**) BSE at 4 keV; (**b**) BSE at 500 eV.

Tuning the information depth by means of the landing energy of electrons may be considered the most straightforward, if not trivial application of the SLEEM method. However, very thin surface layers in units and tens of nm in thickness stop to be fully transparent and hence start contributing to the image signal only at energies well below 1 keV that are usually not available in conventional SEM devices without any kind of beam deceleration. For this reason, we briefly mention this family of samples, illustrated in Figure 24.

## 6. Conclusions

When immersing the sample in a scanning electron microscope in strong electric field, we faced certain restrictions regarding the sample shape and surface treatment but we gained a totally free choice of the landing energy of electrons and, as a side effect, the possibility of complete acquisition of the backscattered electrons at all energies from units of keV to fractions of eV. These features offer a broad range of enhanced or improved contrast mechanisms and even new contrast mechanisms not activated in traditional instruments. Recently the major producers of electron microscopes have been including in their products the possibility of biasing the sample in the kV range so instrumentation is available for the above described imaging method. Thus, precautions have been made to accelerate a so far very slow accumulation of data collected with the SLEEM method.
